# A Multicentre Study on the Efficacy, Safety and Pharmacokinetics of IqYmune®, a Highly Purified 10% Liquid Intravenous Immunoglobulin, in Patients with Primary Immune Deficiency

**DOI:** 10.1007/s10875-017-0416-4

**Published:** 2017-07-15

**Authors:** Gergely Krivan, Ludmila Chernyshova, Larysa Kostyuchenko, Andrzej Lange, Zoltan Nyul, Beata Derfalvi, Jacek Musial, Anne Bellon, Martin Kappler, Alain Sadoun, Ewa Bernatowska

**Affiliations:** 1 Paediatric Haematology and Stem Cell Transplantation Department, Szent Laszlo Hospital, Budapest, Hungary; 2 Department of Paediatric Infectious Diseases and Clinical Immunology, P.L. Shupyk National Medical Academy for Post-graduate Education, Kiev, Ukraine; 3 West Ukrainian Specialized Children’s Medical Centre, Lviv, Ukraine; 4 Lower Silesian Center for Cellular Transplantation/Institute of Immunology and Experimental Therapy, Polish Academy of Sciences, Wroclaw, Poland; 5 Children’s Department, University of Pécs, Pécs, Hungary; 6 2nd Department of Paediatrics, Semmelweis University, Budapest, Hungary; 7 Alergiczno Internistyczny All-Med Specjalistyczny Osrodek, Krakow, Poland; 8 Clinical Development Department, LFB Biotechnologies, BP 40305, 3 avenue des Tropiques, Les Ulis, 91958 Courtaboeuf Cedex France; 9 Statalpha, Baziège, France; 10 Immunology Clinic Department, Children’s Memorial Health Institute, Warsaw, Poland

**Keywords:** IVIg, Immunoglobulins, Clinical trials

## Abstract

This multicentre, open-label, prospective, single-arm study was designed to evaluate the efficacy, pharmacokinetics, and safety of IqYmune®, a highly purified 10% polyvalent immunoglobulin preparation for intravenous administration in patients with primary immunodeficiency. IqYmune® was administered to 62 patients (aged 2–61 years) with X-linked agammaglobulinemia or common variable immune deficiency at a dose from 0.22 to 0.97 g/kg every 3 to 4 weeks for 12 months with an infusion rate up to 8 mL/kg/h. A pharmacokinetic study was performed at steady state between the 8th and the 9th infusion. A single case of serious bacterial infection was observed, leading to an annualized rate of serious bacterial infections/patient (primary endpoint) of 0.017 (98% CI: 0.000, 0.115). Overall, 228 infections were reported, most frequently bronchitis, chronic sinusitis, nasopharyngitis and upper respiratory tract infection. The mean annualized rate of infections was 3.79/patient. A lower risk of infections was associated with an IgG trough level > 8 g/L (*p* = 0.01). The mean annualized durations of absence from work or school and of hospitalization due to infections were 1.01 and 0.89 days/patient, respectively. The mean serum IgG trough level before the 6th infusion was 7.73 g/L after a mean dose of IqYmune® of 0.57 g/kg. The pharmacokinetic profile of IqYmune® was consistent with that of other intravenous immunoglobulins. Overall, 15.5% of infusions were associated with an adverse event occurring within 72 h post infusion. Headache was the most common adverse event. In conclusion, IqYmune® was shown to be effective and well tolerated in patients with primary immunodeficiency.

## Introduction

Primary immunodeficiencies (PIDs) are a heterogeneous group of inherited diseases predisposing individuals to increased risk of infection. To date, more than 200 types of PIDs have been reported in the literature, but less than 10 of them account for more than 60% of all PID cases [1]. Most types of PIDs are associated with a hypogammaglobulinemia due to impaired antibody production. Chronic or recurrent upper and lower respiratory tract infections, sinusitis, and otitis media are the most common infections, while severe bacterial infections (SBIs) such as sepsis, meningitis, septic arthritis, and osteomyelitis can also occur [2–4]. In the absence of early diagnosis and appropriate therapy, recurrent respiratory infections eventually lead to the development of bronchiectases and other chronic pulmonary diseases [5, 6].

Immunoglobulin (Ig) replacement is the mainstay of therapy for PID patients with hypo- or agammaglobulinemia. Its efficacy in preventing severe infections is widely demonstrated [7, 8]. X-linked agammaglobulinemia (XLA) and common variable immune deficiency (CVID) are the most common forms of PIDs requiring Ig replacement therapy [9, 10].

Treatment with intravenous immunoglobulin (IVIg) is generally well tolerated. Headache, chills, fever, and myalgia are the most common adverse reactions, usually mild in intensity [11]. Severe complications are rare and include acute renal failure, mainly associated with sucrose when used as a stabilizer [12], haemolysis caused by the presence of red blood cell IgG alloantibodies in Ig preparations [13], thromboembolic events mainly due to the increased blood viscosity early after Ig administration and/or the presence of pro-coagulant contaminants in Ig preparations [14], and severe allergic reactions, particularly in IgA-deficient patients [15].

IqYmune® is a highly purified 10% liquid preparation of human normal immunoglobulin for intravenous administration obtained from thousands of healthy donors. The manufacturing process consists of cold ethanol and caprylic acid fractionation steps followed by purification steps including an anion exchange chromatography for IgA and IgM clearance and an affinity chromatography ensuring a low anti-A and anti-B haemaglutinin content. The inactivation/removal process of potential blood-borne pathogens is mainly based on a solvent/detergent treatment followed by a 20-nm nanofiltration.

The present study investigated the efficacy, pharmacokinetics, and safety of IqYmune® in paediatric and adult patients with XLA or CVID.

## Clinical Study Methods

### Study Design

An open-label, prospective, single-arm study designed according to the European Medical Agency (EMA) guidelines [16] was conducted in 18 centres in five countries in Europe (France, Hungary, Poland, Serbia and Ukraine) from August 2011 to March 2013. Approvals were obtained from the respective national and institutional ethics committees. Written informed consent and/or assent as appropriate were signed by all patients and/or their legal representatives/witnesses.

### Study Patients

Patients aged from 2 to 65 years with XLA or CVID diagnosed according to the European Society for Immunodeficiencies (ESID) criteria, either Ig-naïve or previously treated with Ig replacement therapy, were recruited. Previously treated patients were to be administered with a stable dose of Ig and to have at least three IgG trough levels ≥4 g/L within the last 6 months prior to study entry.

The main exclusion criteria were history of allergy or serious adverse reaction to Ig therapy, anti-IgA antibodies, glomerular filtration rate (GFR) <80 mL/min/1.73 m^2^ according to MDRD formula in adults or creatinine clearance <60 mL/min/1.73 m^2^ according to Schwartz formula in paediatric patients, alanine aminotransferase (ALT), or aspartate aminotransferase (AST) >3 times upper limit of normal, total bilirubin >2 times upper limit of normal, protein-losing enteropathy or nephrotic syndrome, history of thrombosis within the past 12 months, pregnancy, and breastfeeding.

### Study Product

IqYmune® is a ready-to-use liquid IVIg preparation containing at least 95% of IgG. The IgA content is ≤28 μg/mL, and the osmolality of 300 ± 30 mOsm/kg is in the physiological range. The sodium chloride concentration is <0.02 mmol/L, making the product appropriate for patients with sodium-restricted diet. No procoagulant activity is detected. IqYmune® is stabilized with glycine and polysorbate 80 at a pH of 4.8 ± 0.2 and can be stored at room temperature (25 °C).

### Study Treatments

IqYmune® was to be administered intravenously every 3 or 4 weeks (±3 days) for 12 months, at a stable dose between 0.2 and 0.8 g/kg/month. Dose adjustment was allowed in case of IgG trough level < 6 g/L and/or recurrent infections.

The initial infusion rate was 1 mL/kg/h for 30 min, to be increased up to 4 mL/kg/h for the first three administrations and 8 mL/kg/h for the subsequent infusions.

No premedication was allowed, unless the patient experienced an adverse reaction on two consecutive infusions that could be prevented by acetaminophen, antihistamines, hydroxyzin, non-steroidal anti-inflammatory drugs, or antiemetic agents. Prophylactic antibiotics were forbidden.

### Pharmacokinetics

Serum IgG trough levels were assessed in all patients before each IqYmune® infusion throughout the study period.

In addition, a subset of 28 adult patients (four on a 3-week and 24 on a 4-week dosing schedule) were enrolled in a formal pharmacokinetic (PK) study. Blood samples were drawn shortly before and immediately at the end of the 8th infusion, then 30 min, 6 h, 12 h, 24 h, 3 days, 7 days, 14 days, 21 days and 28 days (if applicable) after the end of infusion. Serum IgG level were measured in a central laboratory using a nephelometric method.

PK parameters were estimated from a population PK model using Phoenix NLME version 1.2. A supportive non-compartmental analysis (NCA) was performed using Phoenix WinNonlin version 6.3 (Pharsight, Cary, NC, USA). The PK parameters assessed were the maximum serum IgG concentration (*C*_max_), the time to reach the maximum serum IgG concentration (*T*_max_), the half-life (*t*_1/2_), the elimination rate constant (*K*_el_), the clearance (Cl), the volume of distribution (Vd), and the area under the concentration-time curve at infinite (AUC_0-∞_).

### Evaluation of Efficacy

The primary efficacy endpoint was the rate of SBIs per patient per year. SBIs included bacterial pneumonia, bacteraemia or sepsis, osteomyelitis, septic arthritis, visceral abscess, and bacterial meningitis as defined in the Food and Drug Administration (FDA) guidance [17].

The secondary efficacy endpoints were the annualized rate of all infections and number of days of infection-related parameters (absence from school/work, hospitalization, fever episode, and antibiotic therapy).

### Evaluation of Safety

Patients were monitored for adverse events (AEs) and serious adverse events (SAEs). Treatment-emergent AEs (TEAEs) were defined as AEs occurring from the time of the first IqYmune® infusion to the date of the end-of-study/early termination (EoS/ET) visit. Temporally associated adverse events (TAAEs) were defined as AEs occurring from the start of infusion up to 72 h after end of infusion.

Vital signs were monitored during and 30 min after the end of each infusion. Biochemistry (AST, ALT, GGT, alkaline phosphatase, total bilirubin, albumin, creatinine, and haptoglobin) and haematology (complete blood count with differential and platelet count) parameters were recorded at baseline, within 30 min after end of each infusion and at the EoS/ET. Direct antiglobulin test (DAT) was analysed at the same time points except EoS/ET.

### Diary Cards

The patients or parents/guardians had to record on diary cards the following information: AEs, infectious episodes and related events (school/work days missed, hospitalisations, and fever), and concomitant medications.

### Statistical Analysis

The statistical analysis was performed on the total treated set (TTS) which consisted of all patients who received at least one IqYmune® administration. All efficacy and safety analyses were performed using SAS^®^ Software version 9.1.3.

Sixty patients were planned to be enrolled in the study to ensure a power of at least 80% to reject a null hypothesis of ≥1 SBI/patient/year, the threshold below which Ig replacement therapy can be considered effective [16]. Assuming that the number of SBIs is following a Poisson distribution, an exact one-sided one-sample Poisson test at a type I error of 0.01 was chosen.

The rate of infusions with at least one TAAE was calculated, and the upper one-sided 95% confidence interval (CI) limit was compared to the threshold of 0.40 specified in the FDA guidance [17].

## Study Results

### Study Patients

A total of 62 patients were enrolled in the study, 36 (58.1%) adults and 26 (41.9%) paediatric patients. Baseline characteristics of the patients are presented in Table 1. Among them, 58 (93.5%) patients were previously on Ig replacement therapy, while four (6.5%) patients were naïve to Ig therapy. Seven (11.3%) patients had a medical history of bronchiectasis. Mean interval from diagnosis of PID to study entry was 7.4 years.

**Table 1:** Patient baseline characteristics (TTS, *N* = 62)

Characteristics	Paediatrics (*n* = 26)	Adults (*n* = 36)	Total (*N* = 62)
Age (years)			
Mean	10.9	39.4	27.4
Range	2–17	18–61	2–61
Gender (*n*, %)			
Male	23 (88.5)	20 (55.6)	43 (69.4)
Female	3 (11.5)	16 (44.4)	19 (30.6)
Weight (kg)			
Mean	42.4	65.7	55.9
Range	12–88	46–95	12–95
Type of PID (*n*, %)			
XLA	17 (65.4)	3 (8.3)	20 (32.3)
CVID	9 (34.6)	33 (91.7)	42 (67.7)
Treatment (*n*, %)			
IVIg	23 (88.5)	34 (94.4)	57 (91.9)
SCIg	1 (3.8)	0 (0.0)	1 (1.6)
Ig-naïve	2 (7.7)	2 (5.6)	4 (6.5)
SBI within the previous year (*n*, %)	5 (19.2)	3 (8.3)	8 (12.9)

*TTS* total treated set

Nine of the 62 (14.5%) patients, all adults, discontinued prematurely the study. Reasons for and times of discontinuations are summarized in Table 2.

**Table 2:** Reasons for and times of premature discontinuations (TTS, *N* = 62)

Reason for discontinuation	*N* (%)	Time of discontinuation (after infusion number)
Adverse event	4 (6.5)	
Infusion-related reaction^a,b^	1 (1.6)	1^c^
Recurrent neutropenia^b^	1 (1.6)	4
Hodgkin’s disease^d^	1 (1.6)	5
Gastrointestinal disorders and ascites^d^	1 (1.6)	5
Withdrawal of consent	2 (3.2)	7 and 8
Late identification of an exclusion criterion	1 (1.6)	2
Pregnancy	1 (1.6)	6
Omission of the last study visit	1 (1.6)	15

*TTS* total treated set

^a^Consisting of a transient episode of dyspnoea, oropharyngeal pain, and chest pain

^b^Drug-related

^c^Five minutes after the start of the infusion

^d^Not drug-related

### Treatment

All enrolled patients were treated with IqYmune®: 57 patients were on a 4-week and five on a 3-week dosing schedule. Patients received a mean number of 12.4 ± 3.2 infusions (range 1–17) leading to a total of 766 infusions over a mean period of 44.5 ± 11.6 weeks (range 0.1–54.4) at a mean dose of 0.56 ± 0.17 g/kg (range 0.22–0.97, excluding one patient who received 0.01 g/kg due to an infusion-related reaction leading to premature study drug discontinuation).

**Fig. 1:**
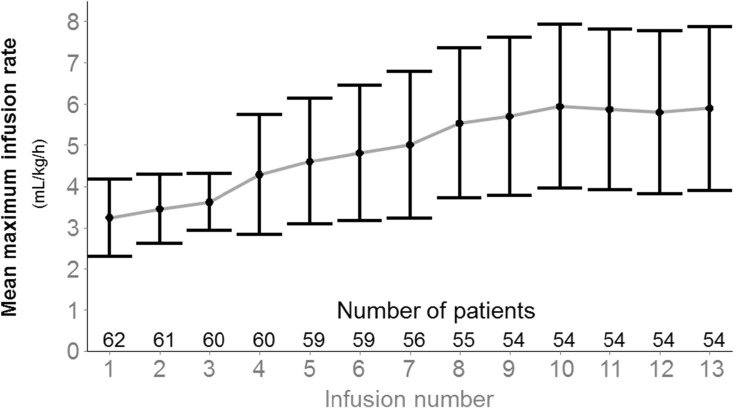
Mean (±SD) maximum infusion rate per infusion over time (TTS, *N* = 62). *SD* standard deviation, *TTS* total treated set. Infusions beyond the 13th are not reported as only five to eight patients were concerned

The mean maximum infusion rate per infusion was 4.88 ± 1.89 mL/kg/h, increasing with the visit number as shown in Fig. 1. The mean maximum infusion rate per patient was 6.10 ± 2.03 mL/kg/h. The maximum infusion rate was ≤4 mL/kg/h for 439 (57.3%) infusions, >4 to 6 mL/kg/h for 162 (21.1%) infusions, and >6 to 8 mL/kg/h for 165 (21.5%) infusions. A total of 27 (43.5%) patients had at least one infusion with a maximum flow rate equal to 8 mL/kg/h. Mean infusion duration was 2.28 ± 0.64 h when the maximum infusion rate was ≤4 mL/kg/h and 1.70 ± 0.40 h when the maximum infusion rate was >4 mL/kg/h.

### Pharmacokinetics

The mean serum IgG trough level in the total population increased from 5.79 ± 2.03 g/L at baseline (range 0.45–9.99) to 7.73 ± 2.36 g/L before the 6th infusion and 7.96 ± 1.53 g/L before the 13th infusion visit (last infusion for patients on a 4-week dosing schedule). In parallel, the mean dose of IqYmune® was 0.48 ± 0.15 g/kg at the 1st infusion, 0.57 ± 0.17 g/kg at the 5th infusion, and 0.60 ± 0.17 g/kg at the 12th infusion. The mean ratio of IgG trough levels over dose by infusion was stable over time and was comparable to that obtained at baseline with the IVIgs administered prior to the first IqYmune® infusion.

The primary PK parameters derived from the population PK model were 4.9 mL/h for clearance and 3.5 L for the volume of distribution in the central compartment. Derived secondary PK parameter estimates were a half-life of 33.6 days and an AUC_0-∞_ of 383 day × g/L. The *C*_max_, calculated using the NCA instead of NLME model to take into account a heterogeneity in infusion durations, was 18.1 g/L. The median *T*_max_ was approximately 2 h after the onset of infusion.

### Efficacy

One SBI was reported during the total study period of 57.74 patient-years, leading to a rate of 0.017 SBI per patient per year significantly lower than the predefined threshold of 1.0 (*p* < 0.001, 98% CI [0.00, 0.115]) as required by the EMA guideline [16].

The only SBI observed was an acinetobacter bacteraemia diagnosed in a 24-year-old male CVID patient just before the 6th IVIg administration. The patient had a serum IgG trough level of 2.47 g/L at the time of the onset of the SBI due to a chronic protein-losing enteropathy gone unnoticed at study entry. Resolution of the infection was complete after intravenous antibiotic therapy.

Overall, a total of 228 infections were experienced by 51 of the 62 patients (82.3%). The mean annualized rate of infections per patient was 3.79 ± 3.62 (range 0.0–14.9) in the total population, 3.01 ± 3.24 (range 0.0–14.0) in adults, and 4.88 ± 3.88 (range 1.0–14.9) in paediatric patients. Most of infections involved the upper respiratory tract. Their distribution is presented in Table 3. Infections were assessed to be mild in severity in 192 cases (84.2%), moderate in 33 cases (14.5%), and severe in 3 cases (1.3%). The severe infections were a gastrointestinal candidiasis and a gastroenteritis cryptosporidial infection in one adult patient and an exacerbation of a chronic sinusitis in one paediatric patient. In all cases, the infections resolved without sequelae.

**Table 3:** Distribution of infections (TTS, *N* = 62)

Infection	Number of patients (% of total patients)	Number of infections (% of total infections)
All infections	51 (82.3)	228 (100)
Bronchitis	15 (24.2)	30 (13.2)
Nasopharyngitis	14 (22.6)	26 (11.4)
Upper respiratory tract infection	11 (17.7)	18 (7.9)
Pharyngitis	10 (16.1)	13 (5.7)
Chronic sinusitis	9 (14.5)	28 (12.3)
Rhinitis	8 (12.9)	12 (5.3)
Tracheitis	7 (11.3)	8 (3.5)
Oral herpes	6 (9.7)	7 (3.1)
Respiratory tract infection	5 (8.1)	5 (2.2)
Conjunctivitis	4 (6.5)	7 (3.1)
Cystitis	4 (6.5)	5 (2.2)
Tracheobronchitis	4 (6.5)	5 (2.2)
Other infections	≤3 (4.8) each	≤4 (1.8) each

*TTS* total treated set

The relationship between infections and IgG trough level (assessed just before the infusion preceding the infection) was analysed. The rate of infusions associated with at least one infection was 26.3 and 18.6% in trough level categories of ≤8 and >8 g/L, respectively (*p* = 0.01).

Other infection-related parameters results (absence from work or school, hospitalization, fever episode, and antibiotics use) are summarized in Table 4.

**Table 4:** Infection-related endpoints (TTS, *N* = 62)

Infection-related endpoints	Number of events	Number of patients (%)	Annualized number of days with events Mean (SD) [95% CI]
All infections	228	51 (82.3)	51.8 (64.6) [35.4, 68.2]
Hospitalization	6	5 (8.1)	0.9 (3.3) [0.1, 1.7]
Fever episode	33	19 (30.6)	1.6 (3.7) [0.6, 2.5]
Antibiotics use	131	38 (61.3)	19.5 (26.8) [12.7, 26.3]
Absence from work or school	15	8 (12.9)	1.0 (3.6) [0.1, 1.9]

*CI* confidence interval, *SD* standard deviation, *TTS* total treated set

### Safety

Adverse events are summarized in Table 5. Fifty-one of the 62 (82.3%) patients experienced a total of 343 TEAEs of mild (79%), moderate (20%), or severe (1%) intensity. Severe TEAEs consisted of one case of anaemia secondary to both inflammation and vitamin B12 deficiency and one case of testicular torsion, and were not related to the study drug.

**Table 5:** Summary of adverse events (TTS, *N* = 62)

	Patients (*n* %) (*N* = 62)	Infusions (*n* %) (*N* = 766)	AEs Total (per infusion)
TEAEs	51 (82.3)	205 (26.8)	343 (0.45)
Drug-related TEAEs	33 (53.2)	105 (13.7)	148 (0.19)
TAAEs	39 (62.9)	119 (15.5)	170 (0.22)
≤4 mL/kg/h (439 infusions)	31 (50.0)	77 (17.5)	116 (0.26)
>4 to ≤6 mL/kg/h (162 infusions)	14 (22.6)	19 (11.7)	24 (0.15)
>6 mL/kg/h (165 infusions)	12 (19.4)	23 (13.9)	30 (0.18)
SAEs	15 (24.2)	19 (2.5)	20 (0.03)
Drug-related SAEs	4 (6.5)	4 (0.005)	4 (0.005)
Discontinuation of study drug due to AE	4 (6.5)	4 (0.005)	5 (0.007)
Interruption of study drug due to AE	4 (6.5)	5 (0.007)	7 (0.009)
Flow rate decrease or no increase due to AE	8 (12.9)	10 (0.03)	11 (0.01)

*AE* adverse event, *SAE* serious adverse event, *TAAE* temporally associated adverse event, *TEAE* treatment-emergent adverse event, *TTS* total treated set

Thirty-three (53.2%) patients presented a total of 148 drug-related TEAEs, most commonly headache (16.1% of patients), chills (14.5%), neutropenia (11.3%), pyrexia (9.7%), back pain (6.5%), and hypertension (6.5%).

Thirty-nine (62.9%) patients experienced a total of 170 TAAEs.

**Fig. 2:**
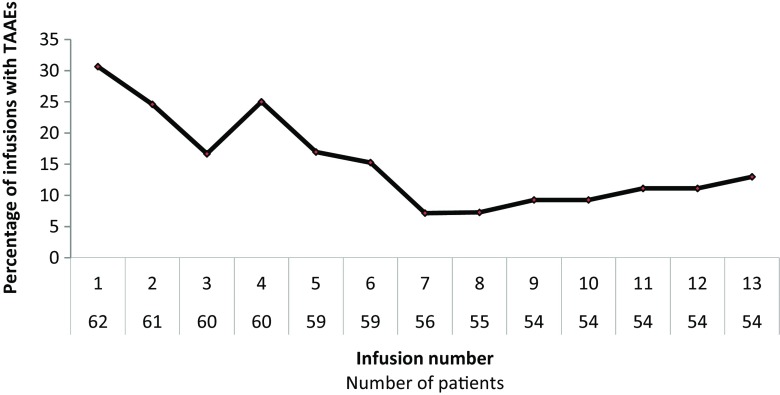
Proportion of infusions with TAAEs over time (TTS, *N* = 62). *TTS* total treated set. Infusions beyond the 13th are not reported as only five to eight patients were concerned

Out of the 766 infusions, 119 were associated with at least one TAAE, resulting in a proportion of 15.5% (95% CI: 13.0; 18.3). As shown in Fig. 2, the percentage of infusions with at least one TAAE was maximal after the first administration and decreased over time. Taking into account the maximum rate of each infusion, 77/439 (17.5%) infusions ≤4 mL/kg/h, 19/162 (11.7%) infusions from >4 to 6 mL/kg/h and 23/165 (13.9%) infusions >6 mL/kg/h were associated with at least one TAAE.

Four (6.5%) patients discontinued the study drug due to a TEAE, including two patients with drug-related TEAEs (see Table 2). One of them presented with a hypersensitivity reaction consisted of an acute infusion-related reaction of moderate intensity with transient dyspnea and oropharyngeal and chest pain. Temporary interruptions and/or flow rate decreases or limitations of an infusion due to a TAAE occurred in 2% of the total infusions, mostly at the first or second administration.

Twenty SAEs were reported in 15 (24.2%) patients including four drug-related SAEs consisting of asymptomatic transient and spontaneously reversible episodes of neutropenia.

A total of 12 (21.1%) of 57 evaluable patients had positive DAT at baseline, whereas 25 (55.6%) of the 45 DAT negative patients converted to positivity during the study period. No patients exhibited biological signs of IVIg-induced haemolysis defined as simultaneous reduction of Hb level ≥ 1 g/dL within 10 days post-infusion and positive DAT associated with low haptoglobin or increased bilirubin serum levels. Regardless of the other parameters associated with hemolysis, no patients presented with a clinically significant low haptoglobin level throughout the study period.

Seven (11.3%) patients were reported with a drug-related neutropenia. Three (3) of them had individual factors potentially contributing to neutropenia, i.e., low neutrophil count at baseline, medical history of bicytopenia, or diagnosis of Hodgkin’s disease during the study period. Return to normal or baseline values was spontaneous at the next pre-infusion analysis in all patients. One neutropenia episode with a nadir <0.5 × 10^9^/L was reported in four of these seven patients. No infections occurred in two of them, while the other two patients presented with mild cystitis and common cold, respectively.

In the whole study population, the mean post-infusion neutrophil count was approximately 1 × 10^9^/L lower than the mean count before the first IqYmune® administration with a greater relative decrease in those patients who had a higher baseline value (data not shown). The ratio of the number of infections over the number of periods with neutrophil count in each categories (<0.5 × 10^9^/L, 0.5 to 1.0 × 10^9^/L, 1.0 to 1.5 × 10^9^/L, and ≥1.5 × 10^9^/L) was similar, ranging between 19.1 and 29.7%. All three severe infections reported in the study were associated with a neutrophil count ≥1.5 × 10^9^/L.

All episodes of hypertension occurred in adults were of mild severity and did not require any medications.

No thromboembolic events, renal failure, or anaphylactic reactions were reported. Analysis of vital signs and laboratory parameters did not show any other safety signals.

## Discussion

The study met its primary efficacy endpoint with an annualized SBI rate of 0.017 per patient significantly lower than the EMA/FDA requirement of less than one SBI/patient/year [16, 17] and in line with SBI rates published in similar studies ranging from 0.0 to 0.12/patient/year [18, 19]. The only SBI occurred in a context of a very low IgG trough level due to an aggravation of a chronic enteropathy with protein loss. The annual rate of all infections of 3.79 per patient and their types were in line with those reported for other IVIg products [18–20]. The study treatment duration of 1 year for a single patient was scheduled in order to prevent a seasonal bias due to a greater rate of infections in the winter months. The higher rate of infections in paediatric patients as compared to adults was expected, due to the exposure to not previously encountered pathogens (mainly viruses) easily transmissible in the childhood environment (child care centres and kindergarten), as well as anatomical ENT characteristics and hypotrophy of mucosal lymphoid tissues.

The number of days of work/school missed due to infections (1.0 day/patient/year) and with curative antibiotic therapy (19.5 days/patient/year) compared favourably with the ranges of 2.28 to 13 days and 32.1 to 55.7 days/patient/year, respectively, reported in the literature while the rate of days of hospitalization (0.9 day/patient/year) was within the range of 0.21 to 2.31 days/patient/year reported with other IVIg products [18–20].

At baseline, the majority of patients (36/62) had IgG trough levels <6 g/L which can be explained by lower Ig doses used in some countries and the inclusion of four Ig-naïve patients. Therefore, doses were increased during the study in order to reach an IgG trough level ≥ 6 g/L and to prevent recurrent infections. Dose increases were associated with proportional increases in IgG trough levels. A satisfactory mean IgG trough level of 7.73 ± 2.36 g/L was achieved before the 6th infusion. As reported in other studies, fewer infections were observed when trough IgG levels were >8 g/L [7, 9, 21, 22]. The mean IgG half-life of 33.6 days was within the range of those reported with other IVIgs [18, 23–25].

IqYmune® was well tolerated. The observed types, severity, and frequency of TEAEs did not differ from those commonly reported with other IVIg preparations [11, 26]. Approximately 99% of the TEAEs were assessed as mild or moderate in severity and the three severe TEAEs were judged as not related to the study drug. As reported in recent studies, both in adults and children, the most frequent TEAE was headache [26, 27]. The rate of infusions with TAAEs of 15.5% was significantly below the safety threshold of 40% required by the FDA [17] and compares favourably with the range of 20.1 to 27.7% recently reported with other IVIg products [18]. Due to the initiation of the IVIg treatment or the switch to a new IVIg preparation, the first infusion was associated with more TAAEs than the subsequent ones [11, 28]. The rate of infusions with at least one TAAE then decreased even though a rebound was observed at the time of the 4th infusion, when maximal infusion rate up to 8 mL/kg/h was first allowed. For the whole study period, the maximum infusion rate was not correlated with the risk of TAAEs (17.5, 11.7, and 13.9% of the infusions with a maximum flow rate of ≤4, 4–6, and >6 mL/kg/h, respectively), showing a good adaptation of the patients to high infusion rates.

Nonspecific positive DAT after IVIg infusions have been reported with an incidence ranging from 8.5 to 47% [18]. This finding is likely to be favoured by the immediate post-infusion sampling and is deemed to be related to the nonspecific binding of IgG to red blood cells [29, 30].

A decrease in neutrophil count is frequently observed with IVIgs. This decrease is of short duration, with a nadir occurring usually within 4 days and a return to baseline within 1 to 2 weeks [31, 32]. Neutrophil count after IVIg depends on the sampling time and blood sampling shortly after the end of each infusion in this study may have favoured the report of neutropenia. Asymptomatic, transient, and spontaneously reversible neutropenia has been reported after IVIg administration in up to 58% of patients, including in patients with PID without an increased risk of infection [32–35]. The pathophysiology of the neutropenia is unclear and several mechanisms have been hypothesised including aggregation of neutrophils [36], adhesion on endothelial cells with rapid migration to tissues [33, 37], and presence of alloantibodies to neutrophil membrane components in the IVIg preparation [38]. IqYmune® tested negative for anti-human neutrophil antigen antibodies. No IgG aggregate or polymer levels were detected that could have activated neutrophils or increase their adhesion to endothelial cells. In addition, no signal of neutrophil activation or degranulation was observed when both the expression of CD11b and lactoferrin were measured ex vivo in a whole blood assay. The absence of severe infections in patients with neutrophil count <0.5 × 10^9^/L is consistent with the assumption that a pool of functionally active blood neutrophils can be mobilized to combat infections.

In summary, IqYmune® is effective in preventing infections and well tolerated as replacement therapy in patients with PID, including at high infusion rates.

## References

[1] Gathmann B, Grimbacher B, Beauté J (2009). The European internet-based patient and research database for primary immunodeficiencies: results 2006–2008. Clin Exp Immunol.

[2] Cooper M. D. (2003). Immunodeficiency Disorders. Hematology.

[3] Wood P (2009). Primary antibody deficiency syndromes. Ann Clin Biochem.

[4] Bonilla FA, Geha RS (2003). Primary immunodeficiency diseases. J Allergy Clin Immunol.

[5] Plebani A, Soresina A, Rondelli R (2002). Clinical, immunological, and molecular analysis in a large cohort of patients with X-linked agammaglobulinemia: an Italian multicenter study. Clin Immunol.

[6] Kainulainen L, Varpula M, Liippo K (1999). Pulmonary abnormalities in patients with primary hypogammaglobulinemia. J Allergy Clin Immunol.

[7] Quartier P, Debré M, De Blic J (1999). Early and prolonged intravenous immunoglobulin replacement therapy in childhood agammaglobulinemia: a retrospective survey of 31 patients. J Pediatr.

[8] Busse PJ, Razvi S, Cunningham-Rundles C (2002). Efficacy of intravenous immunoglobulin in the prevention of pneumonia in patients with common variable immunodeficiency. J Allergy Clin Immunol.

[9] Cunningham-Rundles C (2011). Key aspects for successful immunoglobulin therapy of primary immunodeficiencies. Clin Exp Immunol.

[10] Hernandez-Trujillo HS, Chapel H (2012). Comparison of American and European practices in the management of patients with primary immunodeficiencies. Clin Exp Immunol.

[11] Stiehm ER (2013). Adverse effects of human immunoglobulin therapy. Transfus Med Rev.

[12] Dantal J (2013). Intravenous immunoglobulins: in-depth review of excipients and acute kidney injury risk. Am J Nephrol.

[13] Quinti I, Pulvirenti F, Milito C (2015). Hemolysis in patients with antibody deficiencies on immunoglobulin replacement treatment. Transfusion.

[14] Funk MB, Gross N, Gross S (2013). Thromboembolic events associated with immunoglobulin treatment. Vox Sang.

[15] Anani W, Triulzi D, Yazer MH, Qu L (2014). Relative IgA-deficient recipients have an increased risk of severe allergic transfusion reactions. Vox Sang.

[16] Guideline on the clinical investigation of human normal immunoglobulin for intravenous administration (IVIg). European Medicines Agency: EMA/CHMP/BPWP/94033/2007 rev.2.

[17] FDA Guidance for Industry on safety, efficacy and PK studies to support marketing of human IVGg as replacement therapy for primary humoral immunodeficiency 2008.

[18] Schröder HW, Dougherty CJ (2012). Review of intravenous immunoglobulin replacement therapy trials for primary humoral immunodeficiency patients. Infection.

[19] Kreuz W, Erdös M, Rossi P (2010). A multi-centre study of efficacy and safety of Intratect®, a novel intravenous immunoglobulin preparation. Clin Exp Immunol.

[20] Björkander J, Nikoskelainen J (2006). Prospective open-label study of pharmacokinetics, efficacy and safety of a new 10% liquid intravenous immunoglobulin in patients with hypo- or agammaglobulinemia. Vox Sang.

[21] Orange JS, Grossman WJ, Navickis RJ, Wilkes MM (2010). Impact of trough IgG on pneumonia incidence in primary immunodeficiency: a meta-analysis of clinical studies. Clin Immunol.

[22] Eijkhout HW, Van der Meer JWM, Kallenberg CGM (2001). The effect of two different dosages of intravenous immunoglobulin on the incidence of recurrent infections in patients with primary hypogammaglobulinemia. Ann Intern Med.

[23] Koleba T, Ensom MH (2006). Pharmacokinetics of intravenous immunoglobulin: a systematic review. Pharmacotherapy.

[24] Wasserman RL, Church JA, Peter HH (2009). IgPro10 in PID study group pharmacokinetics of a new 10% intravenous immunoglobulin in patients receiving replacement therapy for primary immunodeficiency. Eur J Pharm Sci.

[25] Ballow M, Notarangelo L, Grimbacher B (2009). Immunodeficiencies. Clin Exp Immunol.

[26] Caress J, Kennedy B, Eickman K (2010). Safety of intravenous immunoglobulin treatment. Expert Opin Drug Saf.

[27] Berger M (2013). Adverse effects of IgG therapy. J Allergy Clin Immunol Pract.

[28] Ameratunga R, Sinclair J, Kolbe J (2004). Increased risk of adverse events when changing intravenous immunoglobulin preparations. Clin Exp Immunol.

[29] Heddle NM, Kelton JG, Turchyn KL, Ali MA (1988). Hypergammaglobulinemia can be associated with a positive direct antiglobulin test, a nonreactive eluate, and no evidence of hemolysis. Transfusion.

[30] Huh YO, Liu FJ, Rogge K (1988). Positive direct antiglobulin test and high serum immunoglobulin G values. Am J Clin Pathol.

[31] Koffman BM, Dalakas MC (1997). Effect of high-dose intravenous immunoglobulin on serum chemistry, hematology, and lymphocyte subpopulations: assessments based on controlled treatment trials in patients with neurological diseases. Muscle Nerve.

[32] Matsuda M, Hosoda W, Sekijima Y (2003). Neutropenia as a complication of high-dose intravenous immunoglobulin therapy in adult patients with neuroimmunologic disorders. Clin Neuropharmacol.

[33] Lemos S, Jacob CMA, Pastorino AC, Castro APBM, Fomin ABF, Carneiro-Sampaio MMS (2009). Neutropenia in antibody-deficient patients under IVIG replacement therapy. Paed Allergy Immunol.

[34] Chae MH, Park SW, Jeon IS (2013). An analysis of neutropenia after the administration of high-dose intravenous immunoglobulin or anti-D immunoglobulin on acute immune thrombocytopenic purpura children: age based analysis. Clin Pediatr Hematol Oncol.

[35] Niebanck AE, Kwiatkowski JL, Raffini LJ (2005). Neutropenia following IVIG therapy in pediatric patients with immune-mediated thrombocytopenia. J Pediatr Hematol Oncol.

[36] Zeltser D, Fusman R, Chapman J (2000). Increased leukocyte aggregation induced by gamma-globulin: a clue to the presence of pseudoleukopenia. Am J Med Sci.

[37] Casulli S, Topçu S, Fattoum L (2011). A differential concentration-dependent effect of IVIg on neutrophil functions: relevance for anti-microbial and anti-inflammatory mechanisms. PLoS One.

[38] Baxley A, Akhtari M (2011). Hematologic toxicities associated with intravenous immunoglobulin therapy. Int Immunopharmacol.

